# Single-chamber Versus Dual-chamber Implantable Cardioverter Defibrillators: Do We Need Physiologic Pacing in The Course?

**Published:** 2006-07-01

**Authors:** Marco Budeus, Thomas Buck, Heinrich Wieneke, Raimund Erbel, Stefan Sack

**Affiliations:** Department of Cardiology, West-German Heart Centre, University of Duisburg-Essen, Germany

**Keywords:** brain natriuretic peptide, 6 minute walk test, single chamber ICD, dual chamber ICD

## Abstract

**Background:**

Many patients with ICD receive different antiarrhythmic drugs (e.g. sotalol, amiodarone, β-blockers) because of ventricular or atrial tachycardias. These drugs can cause AV-block or chronotropic incompetence resulting in a higher percentage of ventricular pacing.

**Methods:**

We analyzed in a retrospective study the impact of DDD(R) versus VVI(R) mode on subjective (NYHA classification) and objective parameters [brain natriuretic peptide (BNP), 6 minute walk test, echocardiography] in 12 of 120 patients (age 60.2 ± 11.2 years; 10 males, 2 females) who needed an upgrading of a single to a dual chamber ICD. The ICD had to be upgraded because of chronotropic incompetence in all patients with signs of progressing heart failure. Data were collected in VVI(R)-pacing and after 6 and 12 months in DDD(R)-pacing with a long AV-interval and AV hysteresis to reduce ventricular pacing.

**Results:**

The 6 minute walk test (392.4 ± 91.4 vs. 324.6 ± 93.3 m, P < 0.001), NYHA-classification (1.4 ± 0.3 vs. 2.6 ± 0.8, P < 0.0001), BNP (234.1 ± 73.5 vs. 410.4 ± 297.0 pg/ml, P < 0.001), left ventricular ejection fraction (49.8 ± 9.6 vs. 36.5 ± 10.9 %, P < 0.0001) and A-wave (73.6 ± 13.7 vs. 41.0 ± 14.0 cm/sec, P < 0.0001) improved with DDD(R)-pacing after 12 months. The ventricular pacing decreased (84.2 ± 18.1 vs. 1.1 ± 1.7 %, P < 0.0001) after 12 months by DDD(R)-pacing with long AV-interval (220.0 ± 10.4 ms) and AV hysteresis.

**Conclusion:**

Our data show a superiority of DDD(R) mode versus VVI(R) mode regarding subjective and objective parameters as NYHA-classification, BNP, 6 minute walk test, left ventricular ejection fraction and left ventricular endsystolic volume after 12 months. The improvements seem to depend on the reduction of ventricular pacing with advanced atrial contraction. But only a small number of patients needed the upgradation.

## Introduction

The main function of implantable cardioverter defibrillator (ICD) therapy is to preserve life by terminating life-threatening tachycardias like ventricular fibrillation (VF) and ventricular tachycardia (VT). Many studies showed the benefit for ICD in primary and secondary prevention of sudden cardiac death in patients with different cardiac diseasess [1-9]. In addition ICD is superior to antiarrhythmic therapy for preventing sudden cardiac death [1,3,5,7,8]. One side effect of ICD therapy is the painful shock needed to terminate the life-threatening tachycardias. Different studies showed a reduction of the ICD therapy by an additional antiarrhythmic therapy [10-14].

Patients having a reduced left ventricular function had worsening of their cardiac function with higher percentage of ventricular pacing [15-18]). Physiologic pacing or no pacing has to be preferred in patients with lower cardiac function and ICD implantation because of worsening of their cardiac function by ventricular pacing. But the accompanying antiarrhythmic therapy (e.g. sotalol, amiodarone, β-blockers) can cause an AV-block or a chronotropic incompetence resulting in a higher percentage of ventricular pacing. Therefore patients with accompanying antiarrhythmic therapy for preventing painful shocks should be paced physiologically or not  paced at all to prevent a worsening of their cardiac function.

The aim of the retrospective investigation was to compare DDD(R) versus VVI(R) pacing on subjective (NYHA classification) and objective [brain natriuretic peptide (BNP), 6 minute walk test, echocardiography] parameters in patients after a required upgrading of a single to a dual chamber ICD for physiologic pacing.

## Methods

We examined 124 patients with different heart diseases and implanted single chamber ICD since 1998 in our study. The ICD was implanted because of a VT (42 patients), VF (59 patients) or a primary indication (23 patients) due to the MADIT II criteria [4]. The ICD had to be implanted at least six months before the inclusion into our study without a pacing indication at the time of the implant. The ICD was programmed to 50 ppm in the VVI mode at the beginning of our retrospective study. We used the interrogable data of the ICD to look for the percentage of ventricular pacing every three months.

We monitored every patient who had an increase of NYHA classification for the indication of upgrading the ICD since 2000 with echocardiography and ECG. The indication for a resynchronization therapy was an intrinsic QRS ≥ 120ms and an asynchrony on echocardiography.

The indication for upgrading to a DDD-ICD was a chronotropic incompetence and a high percentage of ventricular pacing (>70%) corresponding with an increase of NYHA classification. Patients (12 patients) with a chronotropic incompetence and >70% ventricular pacing were programmed to VVIR pacing. When the patients (12 patients) were still in the increased NYHA classification the ICD was upgraded to a DDD-ICD.

Patients were excluded in case of an indication for resynchronization therapy (16 patients), second (no patient) or third (no patient) degree AV block, an inability to walk (no patient) or a life expectancy below 6 months (no patient). The inclusion criterion was an upgradation from single to dual-chamber ICD because of the indication of pacing like chronotropic incompetence. In addition the medication had to be unchanged for 3 months.

Demographic data, medications and medical history were gathered by a patient interview and the review of their medical record at baseline and after six and twelve months. The DDD-ICD was programmed to 50 ppm with rate-responsive pacing, an AV-interval which was 20 ms longer than the intrinsic AV-interval and an AV hysteresis for reducing ventricular pacing (DDDR-50; n = 12). The interrogable data of the ICD were analysed for the percentage of ventricular pacing every three months.

The subjective (NYHA classification) and objective parameters [brain natriuretic peptide (BNP), 6 minute walk test, echocardiography] were evaluated at baseline (within one week before upgrading to a dual-chamber ICD) after six and twelve months in DDD(R)-pacing which were routine clinical practice in our clinic. The 6-minute walk tests were performed randomly during working hours at baseline, after 6 and 12 months. Patients were instructed to walk as far as possible within 6 minutes with a running wheel, with standardized encouragement and breaks when necessary. At the time of echocardiographic evaluation the physician was blinded for the ICD pacing mode. BNP measurement (Triage Meter Plus®, Biosite GmbH, Willich, Germany) was performed randomly during working hours at baseline, after 6 and 12 months.

### Echocardiography

The physicians were blinded to the mode of the ICD during the study. Biplanar left ventricular end-diastolic and end-systolic cavity volumes were calculated using Simpson's rule [19] from paired apical four-chamber and apical long-axis echocardiographic images of a minimum of five cardiac cycles; mean values of each variable were estimated. Biplanar ejection fractions were calculated as End-diastolic volume - End systolic volume / End-diastolic volume x 100% [22]. Pulsed Doppler analysis of mitral inflow included measurements for maximal E and A velocities, E/A ratio. The mean of five measurements was taken as the result. Doppler colour flow mapping was used to identify the presence or absence of mitral valve regurgitation. Gain settings were optimized by reducing the gain to the point where background noise disappeared. The direction of the MR jet was assessed from both parasternal and apical views, and the area of the largest clearly definable colour flow disturbance was traced in each view as an index of the severity of mitral valve regurgitation [21]. Left atrial size was measured by M-mode and two-dimensional echocardiography in all patients with VVI(R) pacing and 6 months after DDD(R) pacing using the Phillips ultrasonic device (3.5 MHz; model Sonos 5500, Philips Medical System, Andover, Massachusetts, USA).

### Statistics

All data are presented as mean values ± standard deviation and as percentages for categorical variables. Data sets were tested with regard to normal distribution. For comparison between baseline and 6 months follow-up, the two-sided Friedman ANOVA test was used for NYHA classification, echocardiographic parameters, 6-minute walk test, BNP and ventricular pacing. A measurement of the linear association between two variables was evaluated using Pearson correlation coefficient. A multivariate Cox regression analysis was performed on variables found to be significant predictors (p < 0.1) with an univariate analysis for upgrading of the ICD. All statistical tests were two-tailed. A P value < 0.05 was considered as statistically significant. SPSS 12.0 for Windows was used as the statistical package.

## Results

The ICD had to be upgraded because of chronotropic incompetence in 12 patients. These patients had a higher incidence of amiodarone therapy (Table 1). Defibrillator systems were manufactured by Biotronik GmbH & Co (Berlin, Germany), Guidant Corp. (St. Paul, MN), Medtronic Inc. (Minneapolis, MN), (alphabetical order). The ICD was implanted because of a VT (8 patients), VF (2 patients) or a primary indication (2 patients) due to the MADIT II criteria [4] without indication for a DDD-ICD at implantation. The clinical characteristics of the patients are shown in Table 1.

The patients received an additional antiarrhythmic therapy in the follow-up because of paroxysmal atrial fibrillation with inappropriate shocks (3 patients) and repeated VTs with appropriate shocks (9 patients). Paroxysmal atrial fibrillation was no longer observed during the further follow-up after the additional antiarrhythmic therapy with amiodarone in the three patients with inappropriate shocks.

The increase of NYHA classification occurred 11.6 ± 2.3 months after receiving an additional antiarrhythmic therapy caused by a sinus bradycardia and programming rate-responsive pacing function. The heart rate decreased from 59.0 ± 12.7 to 31.2 ± 5.2 (P < 0.0001) due to the antiarrhythmic therapy with amiodarone. The ICD had to be upgraded after a mean follow-up of 25.8 ± 13.7 months after ICD implant and nearly 85% ventricular pacing in all patients. The programmed AV-interval was 220.0 ± 10.4 ms.

A retrograde ventricular-atrial conduction was excluded with the dual chamber ICD as reason for the impaired effect of VVIR-and VVI-pacing.

### Subjective parameters

The NYHA classification increased significantly from 1.5 ± 0.4 at implant to 2.6 < 0.8 nearly 12 months with >70% ventricular pacing after receiving amiodarone therapy. After 6 months (1.6 ± 0.6 vs. 2.6 < 0.8; P ± 0.0001) and after 12 months (1.4 ± 0.3 vs. 2.6 < 0.8, P < 0.0001) with DDD(R) pacing NYHA classification decreased significantly.

### Objective parameters

After 6 months with DDD(R) pacing the BNP (410.4 ± 297.0 vs. 312.3 ± 213.6 pg/ml, P < 0.014) and 6 minute walk test (324.6 ± 93.3 vs. 374.7 ± 113.2 m, P < 0.013) improved in comparison to VVI(R) pacing. The 6-minute walk test (392.4 ± 91.4 vs. 324.6 ± 93.3 m, P < 0.001), and BNP (234.1 ± 73.5 vs. 410.4 ± 297.0 pg/ml, P < 0.001) improved further after 12 months.

The left ventricular endsystolic volume, left ventricular ejection fraction, left atrial size (and A-wave also improved significantly after 6 and 12 months with DDD(R) pacing (Table 2). The ventricular pacing was significantly reduced from 84.2 ± 18.1% to 1.1 ± 1.7  % (P < 0.0001) after 12 months with DDD(R) pacing. During the study the diuretic therapy was reduced in 8 (75%) patients. This reduction included a reduction of furosemide (67.5 ± 30.1 vs. 40.0  ± 28.9 mg, p < 0.054) and spironolactone (65.6 ± 22.9 vs. 37.5 ± 24.9 mg, p < 0.018). In the other four patients the medication was unchanged.

### Correlation

We observed positive correlations between the reduction of ventricular pacing and improvement at the 6 minute walk test (r = 0.84, P < 0.001), NYHA classification (r = 0.79, P < 0.001), the improvement of the BNP (r = 0.62, P < 0.031), the left ventricular endsystolic volume (r = 0.69, P < 0.019) and left ventricular ejection fraction (r = 0.92, P < 0.001).

In the multivariate regression analysis amiodarone treatment (odds ratio 31.6; 95% Cl 4.26-122.05; p < 0.0001) and a ventricular pacing > 10% (odds ratio 129.7; 95% Cl 19.28-315.73; p < 0.0001) were independent parameters for chronotropic incompetence corresponding with the upgrading to a DDD-ICD.

## Discussion

In the present study we observed a significant improvement of subjective (NYHA classification) and objective (BNP, 6 minute walk test, echocardiography) parameters after 12 months in DDD(R)-pacing compared with VVI(R)/VVI-pacing. The improvements correlated with a reduction of ventricular pacing. The additional implantation of an atrial lead and a programmed long AV-interval and AV hysteresis caused a reduction of ventricular pacing. We believe that the improvement was caused by atrioventricular synchrony and a larger part by  reduction of ventricular pacing.

### Comparison of VVI vs DDD pacing

All patients  received a single chamber ICD for primary or secondary prevention of sudden cardiac death. The ICD had to be upgraded because of nearly 100% ventricular pacing corresponding with an increase of NYHA classification in our study. Former studies also observed an unanticipated necessity of pacing during the follow-up [22,24]. We showed an improved of NYHA-classification, BNP, 6 minute walk test, left ventricular ejection fraction and left ventricular endsystolic volume after 6 and 12 months with DDD(R)-pacing compared with VVI(R)-pacing. The improvements were significantly correlated with the reduction of ventricular pacing. On the other side left atrial contraction was improved with physiologic pacing which became apparent by an increased A wave and the reduction of left atrial size.

The DAVID Trial confirmed the disadvantage of DDD(R)-pacing regarding a higher mortality and hospitalization for congestive heart failure whether ventricular pacing was  necessary or not [15]. Because of the high incidence of ventricular pacing (55.7%) the left ventricular function worsened in the DAVID Trail due to programmed rate-responsive pacing at 70/min for DDD pacing [15-18].

In our study the improvements were achieved by a reduction of ventricular pacing. The histological alterations [25,26] as a result of ventricular pacing were shown by myofibril disarray [25,26] followed by an impaired cardiac function DDD pacing [15-18].

### Echocardiography

The advantage of physiologic pacing was shown by different echocardiographic parameters. By means of synchrony of atrial and ventricular contraction a reduction of left ventricular endsystolic volume and left atrial size was accomplished DDD pacing [27]. In addition the increase of the A wave suggested an improved left atrial contraction with physiologic pacing DDD pacing [15,29]. As a result of these improvements we observed an increase of the left ventricular ejection fraction like former studies DDD pacing [27-31].

VVI pacing results in an impairment of the left ventricular function because of a loss of atrioventricular synchrony in patients with heart failure [27,29,31]. or by the pacemaker syndrome [32]. The loss of the atrioventricular synchrony is reversible in time due to physiologic pacing [28]. These parameters can be evaluated by echocardiography, which is an appropriate examination for the course of patients with pacemaker or ICD.

## Limitation

The examination of a small study sample reduced the statistical significance of our results. In addition this was a short-term observational study and long-term outcomes are unknown in our trial. But similar results can be found in former studies with larger patients sample size [15-18,22-24,27-31]. The influence of DDD(R) pacing on the incidence of atrial or ventricular tachyarrhythmias can not be estimated because the observation period between DDD(R) and VVI(R) pacing was too short. We could not fully achieve the blinding of the echocardiographer because the additional lead was visible in the right atrium and the difference between VVI pacing and AV synchrony was also apparent.

## Conclusions

The results of our study suggest that physiologic pacing improved subjective (NYHA classification) and objective (BNP, 6 minute walk test, echocardiography) parameters of patients with a new pacing indication. Physiologic pacing improves atrial and ventricular function in comparison to VVI(R) pacing. The reduction of ventricular pacing was the reason for these improvements. Even though the attention has been directed towards the cardiac resynchronization therapy for the improvement of heart failure we could achieve a significant enhancement of heart failure due to a reduction of ventricular pacing with increased physiologic pacing with a long AV-interval. In addition the indication for upgrading to a dual chamber ICD was accomplished in a small group of patients (10%). Thus in the most cases the implantation of a single chamber ICD was adequate. But this problem is going to accompany the clinical work. Amiodarone seems to be the risk factor for the upgrading of the ICD according to high incidence of ventricular pacing because of its antitachycardia effect.

## Figures and Tables

**Table 1:**
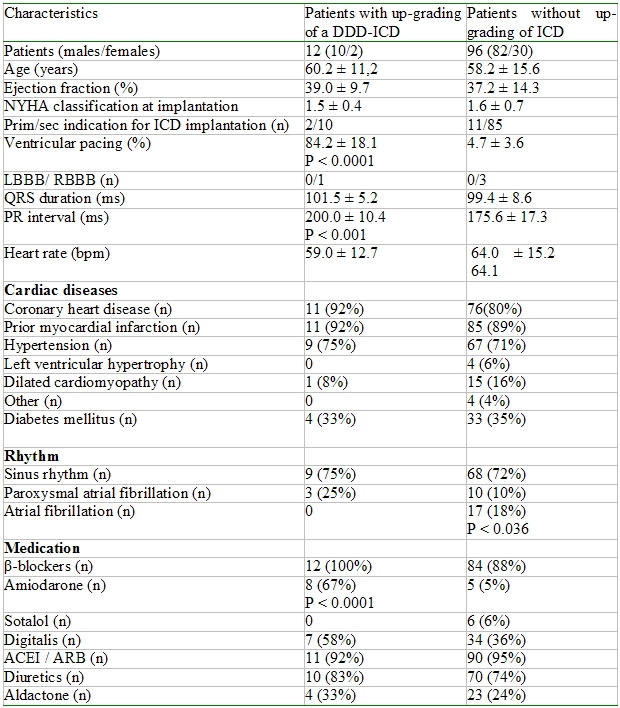
Patients characteristics

**Table 2:**
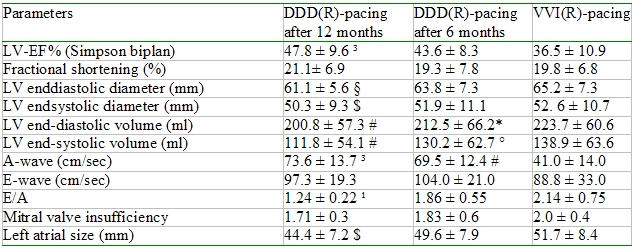
Comparison of echocardiographic results

# = p < 0.001 in comparison to VVI(R)-pacing, * = p < 0.082 in comparison to VVI(R)-pacing, ° = p < 0.037 in comparison to VVI(R)-pacing, ^1^ = p < 0.005 in comparison to VVI(R)-pacing, ^2^ = p < 0.025 in comparison to VVI(R)-pacing, ^3^ = p < 0.0001 in comparison to VVI(R)-pacing, § = p< 0.011 in comparison to VVI(R)-pacing, $ = p < 0.05 in comparison to VVI(R)-pacing
